# Promising Antifungal Activity of *Encephalartos laurentianus* de Wild against *Candida albicans* Clinical Isolates: In Vitro and In Vivo Effects on Renal Cortex of Adult Albino Rats

**DOI:** 10.3390/jof8050426

**Published:** 2022-04-21

**Authors:** Walaa A. Negm, Mona El-Aasr, Ghada Attia, Moneerah J. Alqahtani, Rania Ibrahim Yassien, Amal Abo Kamer, Engy Elekhnawy

**Affiliations:** 1 Department of Pharmacognosy, Faculty of Pharmacy, Tanta University, Tanta 31527, Egypt; moelaasar@pharm.tanta.edu.eg (M.E.-A.); ghada.attia@pharm.tanta.edu.eg (G.A.); 2 Department of Pharmacognosy, College of Pharmacy, King Saud University, Riyadh 11451, Saudi Arabia; mjalqahtani@ksu.edu.sa; 3 Department of BioMolecular Sciences, Division of Pharmacognosy, School of Pharmacy, University of Mississippi, Oxford, MS 38677, USA; 4 Department of Histology and Cell Biology, Faculty of Medicine, Menoufia University, Shebin El-Kom 32511, Egypt; raniayassien@yahoo.com; 5 Pharmaceutical Microbiology Department, Faculty of Pharmacy, Tanta University, Tanta 31527, Egypt; amalabokamer@pharm.tanta.edu.eg

**Keywords:** alkaline phosphatase, desmin, fluorescent microscope, iNOs, LC-MS/MS, SEM, qRT-PCR

## Abstract

*Candida albicans* can cause various infections, especially in immunocompromised patients. Its ability to develop resistance to the current antifungal drugs as well as its multiple virulence factors have rendered the problem even more complicated. Thus, in the present investigation, we elucidated an in vitro and in vivo antifungal activity of *Encephalartos laurentianus* methanol extract (ELME) against *C. albicans* clinical isolates for the first time. A phytochemical identification of 64 compounds was conducted in ELME using LC-MS/MS. Interestingly, ELME exhibited antifungal activity with MIC values that ranged from 32–256 µg/mL. Furthermore, we investigated the antibiofilm activity of ELME against the biofilms formed by *C. albicans* isolates. ELME displayed antibiofilm activity using a crystal violet assay as it decreased the percentages of cells, moderately and strongly forming biofilms from 62.5% to 25%. Moreover, the antibiofilm impact of ELME was elucidated using SEM and fluorescent microscope. A significant reduction in the biofilm formation by *C. albicans* isolates was observed. In addition, we observed that ELME resulted in the downregulation of the biofilm-related tested genes (*ALS1*, *BCR1*, *PLB2*, and *SAP5*) in 37.5% of the isolates using qRT-PCR. Besides, the in vivo antifungal activity of ELME on the kidney tissues of rats infected with *C. albicans* was investigated using histological and immunohistochemical studies. ELME was found to protect against *C. albicans* induced renal damage, decrease desmin and inducible nitric oxide synthase, increase alkaline phosphatase, and increase infected rats’ survival rate. Additionally, the cytotoxicity of ELME was elucidated on Human Skin Fibroblast normal cells using MTT assay. ELME had an IC_50_ of 31.26 µg/mL. Thus, we can conclude that ELME might be a promising future source for antifungal compounds.

## 1. Introduction

In recent decades, *Candida* species have been considered a common cause of nosocomial infections, leading to high morbidity and mortality rates. *Candida* is capable of causing both superficial (mucocutaneous and cutaneous) and life-threatening invasive infections such as candidemia [1]. There are many species of *Candida* such as *C. albicans*, *C. krusei*, *C. glabrata*, *C. parapsilosis*, and *C. tropicalis*. However, *C. albicans* is the most commonly isolated species. *C. albicans* is a harmless commensal fungus commonly present in the oral cavity and vagina, but, upon appropriate conditions, it can be converted to be pathogenic fungus [2].

*C. albicans* have many virulence factors that enable them to overgrow and invade the host tissues causing different infections such as its ability to express adhesins and secretion of hydrolytic enzymes. However, the most important virulence factor is its ability to form biofilms. It is a characteristic that allows them to adhere to different surfaces, biotic and abiotic [3]. Biofilms are large communities of adjacent cells embedded in an extracellular matrix. Clinically, biofilms are difficult to be treated owing to the resistance to antimicrobials and human defences [4].

There is an increased incidence of invasive infections caused by *C. albicans,* especially in immuno-compromised patients such as cancer and AIDS patients. This is accompanied by the development and dissemination of resistance against most conventional antifungal agents [5]. In addition, the currently present antifungal agents suffer from many disadvantages such as the narrow spectrum of activity, low potency, high cost, and multiple adverse effects. Thus, there is an urgent need to find out and develop new therapeutics against the infections caused by *C. albicans* [6]. 

In recent decades, natural products represented a significant starting point for the therapeutic compounds that can treat various diseases. In different regions of the world, we can find that several groups of researchers have studied the biological activity of plants [7]. For example, many plants have shown significant antimicrobial activity (both in vitro and in vivo), a finding that has led to a more intense search for the antimicrobial activity of many plants [8,9]. Plants contain several active constituents that effectively control the growth of different microorganisms, including fungi [10]. There are various secondary metabolites in plants such as alkaloids, tannins, flavonoids, terpenoids, glycosides, etc., that have in vitro antimicrobial potential [11].

*Encephalartos*, the second biggest extant genus of the Cycadales, is restricted to Africa south of the Sahara, with roughly 66 species. The tropical regions of central and east Africa have many species, but South Africa has more than half of them. They are endemism-rich and occupy a wide range of climate regimes and habitats [12].

*Encephalartos laurentianus* De Wild. (Family Zamiaceae), often known as the malele or Kwango giant cycad, it is a cycad species found in northern Angola and southern Congo. It is the hugest of all the cycads. *E. laurentianus* was recently found to have cytotoxic, antioxidant, and antimicrobial properties [13,14].

In the current study, we aimed to investigate the antifungal activity of *E. laurentianus* methanol extract (ELME) against *C. albicans* clinical isolates, both in vitro and in vivo. In addition, the phytochemical constituents of ELME were analyzed using LC-ESI-MS/MS technique.

## 2. Materials and Methods

### 2.1. Chemicals and Plant Materials

*E. laurentianus* De Wild. leaves (four years old tree) were gathered from Al Abid Farms, Giza Governorate on 13 January 2017. The plant was recognized by Dr. Esraa Ammar, Faculty of Science, Tanta University. A voucher sample (PGG-003-W) was deposited at the Herbarium of the Pharmacognosy Department, Tanta University. The plant was dried for 14 days at room temperature at 25° ± 2, then for 2 days in the oven at 40°. The dried leaves were then reduced and ground to a fine powder. The powder (250 g) was extracted with methanol (2.5 L × 4 times) using the cold maceration method to yield 26.7 g of ELME. All of the other chemicals used in the current study were obtained from Merck, Kenilworth, NJ, USA. 

### 2.2. Isolation and Identification of C. albicans Isolates

In the current study, 16 *C. albicans* clinical isolates were collected from different patient specimens from the clinical laboratories of Tanta University Hospitals, Egypt. The samples were cultured on sabouraud dextrose agar (SDA) plates (Oxoid, Basingstoke, UK), and they were overnight incubated at 37 °C. Then, *Candida* isolates were cultured on HiCrome™ *Candida* differential agar (Himedia, Mumbai, India) using *Candida albicans* (MTCC 227) as standard strain. In addition, the API 20C system (BioMérieux, Craponne, France) was used according to the manufacturer’s instructions. 

### 2.3. Animals

A total of 75 adult male albino rats weighing 170–200 g of three months of age were used in the current work. The rats were adequately housed ventilated cages with controlled temperature (25 °C), humidity, and 12 h light/dark cycles. In addition, they were allowed free access to water and food. Strict hygiene was followed to keep a healthy medium for them. All of the animal protocols were authorized and observed via the Animal Care Committee of the Research Laboratory of Experimental Animals at the Faculty of Medicine, Menoufia University, Egypt, with approval number 2/2022OBSGN. 

### 2.4. LC-MS/MS for Metabolite Profiling

The LC-ESI-MS/MS analysis was conducted at the Children’s Cancer Hospital’s Proteomics and Metabolomics Unit (57357). We adopted the criteria has been previously described [15,16]. 

Secondary metabolites of the ELME were analyzed using an ExionLC -High flow LC, Sciex^®^, Framingham, MA, USA UPLC analytical technique, combined with a Triple TOF. 5600+ (Sciex^®^) for IDA. acquisition and Analyst TF 1.7.1 (Sciex^®^) for LC-Triple TOF. control. After the filtration process by an in-line filter disks (0.5 μm × 3.0 mm, Phenomenex^®^, Torrance, CA, USA), 1 g/mL of ELME was injected into X Select HSS. T3 XP UHPLC column (2.5 μm, 2.1 × 150 mm, Waters^®^, Milford, MA, USA) at 40 °C, and eluted using buffer systems of 1% methanol in 5 mM ammonium formate buffer at pH 3 and pH 8 as solvent A (for positive mode MS analysis), and B (for negative mode MS analysis), respectively, and 100% of acetonitrile as solvent C at 0.3 mL/min flow rate. Gradient mobile phase mixtures were composed of 90% solvent A or B and 10% of solvent C, which were injected for 20 min, then reversed into 10% of solvent A or B to 90% of solvent C for the next 5 min and finalized by loading of the starting mixture for the last 3 min. The resultant total ion chromatogram (TIC) was used for master view peaks extraction with a signal-to-noise ratio greater than 5 (non-targeted analysis) and more than 3 feature intensities of the sample-to-blank ratio. Data interpretation was accomplished by applying a Reifycs Abf (Analysis Base File) Converter (Reifycs^®^, Tokyo, Japan) for Wiff file conversion and MS-DIAL 4.6 (RIKEN^®^ Tokyo, Japan). 

To identify chemicals, PeakView^TM^ software version 2 1 was used to compare retention duration and m/z values obtained by MS and MS^2^. The XIC Manager in PeakView^TM^ software was used to calculate peak area values. Extracted ion chromatograms (XICs) for each targeted analyte were automatically created and compared to a user-defined threshold [17].

### 2.5. In Vitro Antifungal Activity of ELME

#### 2.5.1. Disc Agar Diffusion

The susceptibility of *C. albicans* isolates to ELME was investigated using the disk agar diffusion method [17,18]. In brief, 3–5 fungal colonies were inoculated into sabouraud dextrose broth (SDB) (Oxoid, Hampshire, UK) and overnight incubated at 37 °C. After adjusting the turbidity of the suspensions to 0.5 McFarland turbidity standard, they were spread on the surface of Muller Hinton agar (MHA) plates (Oxoid, Hampshire, UK), supplemented with 2% glucose 0.5 µg/mL methylene blue, using sterile cotton swabs. Then, sterile filter paper disks, previously loaded with ELME, were placed on the surfaces of MHA plates by sterile forceps, and the plates were incubated at 37 °C for 24 h.

#### 2.5.2. Determination of Minimum Inhibitory Concentration (MIC)

Broth microdilution assay was utilized in the current study to determine the MIC values of ELME against the tested *C. albicans* isolates [18,19] in 96-well microtitration plates. In brief, fungal colonies were inoculated into SDB and incubated overnight at 37 °C in an orbital shaker (New Brunswich, Fredericton, Canada). The fungal cells were centrifuged, the pellets were rinsed twice with phosphate-buffered saline (PBS) and resuspended in Roswell Park Memorial Institute 1640 (RPMI 1640) with a concentration of 1 × 10^3^ CFU/mL. ELME was serially diluted by adding 50 µL and 50 µL of the fungal suspensions. The concentration range of ELME was from 512 to 1 µg/mL. The plates contained positive (fungi only without ELME) and negative (RPMI 1640 only without *Candida*) controls, then they were incubated at 37 °C for 24 h. This test was carried out in triplicates. The lowest concentration of ELME, which inhibited the visible growth of *C. albicans*, was regarded as the MIC and 0.5 MIC values of ELME were used for further studies. 

#### 2.5.3. Biofilm Formation Assay

The b§iofilm formation assay was carried out as previously described [20]. Briefly, overnight *C. albicans* cultures were diluted with SDB and adjusted to 1 × 10^6^ CFU/mL. Then, fungal suspensions (100 μL) were inoculated into 96-well microtitration plates and incubated at 37 °C for 48 h. The wells were gently washed with PBS, and the adherent fungal cells were fixed with 100 μL methanol (99%) for 20 min. They were then stained with crystal violet (1%) for 20 min. After that, the wells of the microtitration plates were washed using distilled water and were left to dry. The dye bound to the fungal cells was dissolved using 33% glacial acetic acid, and their optical density (OD) was recorded at 540 nm using an ELISA reader (Sunrise Tecan, Switzerland, Austria). This assay was carried out in triplicate and cut-off OD (ODc) was calculated. ODc is the mean OD of the negative control (SDB only without fungi) plus three times standard deviations (SD). *C. albicans* isolates were grouped into non-biofilm forming (OD ≤ ODc), weak biofilm forming (OD > ODc ≤ 2 × ODc), moderate biofilm-forming (OD >  2 × ODc ≤ 4 × ODc), and strong biofilm-forming (OD > 4 × ODc).

#### 2.5.4. Antibiofilm Activity of ELME

The antibiofilm activity of ELME was investigated against the moderate and strong biofilm-forming *C. albicans* isolates before and after treatment with ELME at 0.5 MIC values using crystal violet assay as previously described [21]. The viability of the formed biofilms in the microtitration plates was assessed by counting the colony-forming units (CFU/mL). The formed biofilms were gently washed with PBS, and they were then scraped off the microtitration plate wells using pipette tips or toothpicks after adding 200 μL PBS. After that, the obtained suspensions were serially diluted, 100 μL was plated on SDA plates supplemented with chloramphenicol, incubated overnight at 37 °C, and the number of CFU/mL was counted.

a. Scanning electron microscope (SEM)

As previously reported, an inspection of the biofilm morphology of *C. albicans* isolates by SEM was performed [22]. Briefly, glass coverslips were submerged with each *C. albicans* isolates (before and after treatment with ELME), and they were left for 24 h to let the isolates form biofilms. The formed biofilms were gently washed with PBS and flooded with glutaraldehyde solution (2.5%) for 24 h at 4 °C. After that, they were dehydrated by a series of ethanol with concentrations that ranged from 30% to 100%. They were then left to dry and coated with gold for inspection with SEM (Hitachi, Tokyo, Japan).

b. Fluorescent microscope

Glass coverslips with *C. albicans* biofilms were transferred to microscopical slides and stained with a Calcofluor White stain for 2 min in a dark room. Then, biofilms were observed as soon as possible using a fluorescence microscope [23].

c. Quantitative real-time polymerase chain reaction (qRT-PCR)

The impact of ELME on the gene expression of the biofilm genes (*ALS1*, *BCR1*, *PLB2*, and *SAP5*) was assessed in all of the *C. albicans* isolates using qRT-PCR as previously described [24]. In brief, total RNA was extracted using RNeasy mini kit (Qiagen, Hilden, Germany). Then, the extracted RNA was retrotranscribed into cDNA using SensiFAST™ cDNA kit (Bioline, London, UK). qRT-PCR was performed using SensiFAST™ SYBR green PCR master mix (Bioline, London, UK) according to the manufacturer instructions using the primers listed in Table S1. The expression of the tested genes in the fungal cells before treatment was set to be 1. 

### 2.6. In Vivo Antifungal Activity of ELME

#### 2.6.1. Experimental Protocol

All of the animals were exposed to immunosuppression by corticosteroid injection [25]. Then, animals were randomly divided into 5 groups as follows:

Group I (control group): 15 rats each received 1 mL saline (0.9%) by intraperitoneal (IP) injection once daily for 1 week;
Group II (*C. albicans* group, C6 isolate, with the concentration of 2 × 105 CFU/mL): 15 rats each received a suspension of *C. albicans* (0.1 mL) by intravenous (IV) injection in the tail vein once;
Group III (fluconazole treated group): 15 rats each received a suspension of *C. albicans* (0.1 mL) by IV injection in the tail vein once. Then, this group received 10 mg/kg fluconazole by IP injection for 1 week;
Group IV (ELME treated group, 50 mg/kg): 15 rats received a suspension of *C. albicans* (0.1 mL) by IV injection in the tail vein once. Then, this group received 50 mg/kg ELME by IP injection for 1 week;
Group V (ELME treated group, 100 mg/kg): 15 rats received a suspension of *C. albicans* (0.1 mL) by IV injection in the tail vein once. Then, this group received 100 mg/kg ELME by IP injection for 1 week;
Rats were monitored for 15 days after infection to determine the survival rate. Ether inhalation was used to anesthetize all rats to be sacrificed. Kidney tissues were obtained and processed for histological and immunohistochemical investigations. In addition, fungal tissue burden (number of CFU/g kidney tissues) was calculated.

#### 2.6.2. Histopathological and Immunohistochemical Studies

Kidney specimens from all of the animal groups were fixed in normal saline. The specimens were processed for performing paraffin sections, 7 μm in thickness. Then, sections were stained with hematoxylin and eosin (H&E) to illustrate the histological structure, and Masson’s trichrome stain was used to detect the collagen fibers [26]. 

For the immunohistochemical study of desmin, a podocyte injury marker, the utilized primary monoclonal antibody was anti-desmin (Lab Vision Corp, Inc./Lab Vision, Fremont, CA, USA). A negative control was performed via replacing the primary antibody with buffer alone. Uterus smooth muscle (myometrium) was used as a positive control. The positive reaction was cytoplasmic brown color [27]. Counterstaining was performed using Mayer’s hematoxylin.

For the histochemical study, specimens from the renal cortex were fixed in cold acetone, then processed and stained using calcium phosphate according to the Gomori technique to determine the alkaline phosphatase enzyme. The histochemical activity of alkaline phosphatase in the proximal convoluted tubules was detected. The reaction product was strongly marked at the apical surface and the basal part of the cells comprising the basal infoldings with their extended tips. The positive reaction was in fine, uniform brown deposits [28].

For immunostaining of inducible nitric oxide synthase (iNOS), iNOS antibody was utilized, a polyclonal antibody (Lab Vision Corporation Laboratories, catalogue number PA1-036). Immunostaining required pretreatment by boiling for 10 min in 10 Mm citrate buffer, pH 6 for antigen retrieval, and then sections were left to cool at room temperature for 20 min. After that, the sections were incubated for one hour with the primary antibodies and immunostaining was completed using the Ultravision detection system (cat no T.P.-015- H.D.) [29].

#### 2.6.3. Morphometric Measurements

These measurements were carried out for histological and immunohistochemical quantitative assessment. Five non-overlapping fields per animal were randomly captured by a Lieca Microscope D.M.L. B2/11888111 equipped with a Leica camera DFC450. Kidney sections were randomly selected for morphometric measurements using image analyzer software (Image J analyzer version 1.43o8, National Institutes of Health, Bethesda, MD, USA). The following parameters were measured: the glomerular basement membrane (GBM) thickness (×200), the area percentage of collagen fibers stained by Masson trichrome stain (×200) and the area percentage of desmin and iNOs positive immune staining (×400).

Histological damage of the renal tubule was assessed as the percentage of the tubules that appeared dilated, atrophied, tubules with epithelial cell vacuolated cytoplasm, and with cast formation as 0 (normal), 1 (<10%), 2 (10 to 25%), 3 (26 to 50%), 4 (51 to 75%), and 5 (>75%). H&E-stained kidney segments were utilized to examine the renal tubular damage score. Five regions of the renal tubules were randomly chosen per kidney from each of the ten rats in each group for evaluation at a magnification of ×200, and the average score was calculated [30].

### 2.7. Cytotoxicity MTT Assay

The MTT assay was used to determine the cytotoxicity of ELME on human skin fibroblast (HSF) normal cell line (Nawah-Scientific, Cairo, Egypt). The cells were cultured in Dulbecco’s Modified Eagle Medium (DMEM) media containing 10% fetal bovine serum (FBS), 100 units/mL penicillin, and 100 mg/mL streptomycin. The cultures were kept at 37 °C in a humidified environment with 5% CO_2_. Confluent monolayers of cells were cultivated for 24 h in 96 well microtitration plates. Then, cells were cultured with varying doses of ELME in triplicate for 72 h. After that, 20 µL of 5 mg/mL MTT were gently poured into each well and incubated for 4 hours at 37 °C. The media was then removed carefully, and 150 μL of dimethyl sulfoxide (DMSO) solvent was added to dissolve the formed formazan crystals. Finally, using a microplate reader (B.M.G. Labtech, FLUOstar Omega, Ortenberg, Germany), OD values were measured at 540 nm [31].

### 2.8. Statistical Analysis

Data were shown as mean ± standard deviation. The differences between the tested groups were assessed using a one-way analysis of variance (ANOVA), followed by a post hoc test (Tukey). Kruskal Wallis test was used for scoring the tubular injury. In addition, Kaplan-Meier survival curve was used to calculate the survival of rats. The difference is significant at *p* < 0.05 using Prism version 8 (GraphPad Software, Inc., San Diego, CA, USA).

## 3. Results

### 3.1. LC-ESI-MS/MS of ELME

LC-ESI-MS/MS analysis of ELME (negative and positive mode E.S.I.) tentatively identified 64 compounds of secondary metabolites for the first time. The identified metabolites were classified as anthocyanidin-3-*O*-glycosides, alkaloids, aurone derivative, coumarins, flavonoid aglycone, flavonoid glycosides, phenolic, and carboxylic acid. Table 1 shows the results of LC-ESI-MS/MS analysis of ELME metabolites, and Figures S1 and S2 show the total ion chromatogram (TIC) of ELME in positive and negative mode E.S.I., respectively.

#### 3.1.1. Identification of Flavonoids, Biflavonoids, and Catechins

Flavonoids aglycones and their glycosides resemble most of the compounds detected in ELME. The identified flavonoids aglycones were represented by 13 compounds in positive ion mode are (41, 42, 46, 47, 48, 50, 51, 52, 53, 54, 55, 57, 64) with [M + H]^+^ at *m/z* of 289.07, 287.04, 273.07, 301.10, 317.06, 269.07, 287.09, 285.08, 271.09, 287.09, 305.13, 289.18, 317.11, respectively. Apigenin (53) was the major detected flavonoid compound in positive ion mode according to peak area measurements with [M + H]^+^ at *m/z* of 271.09 and characteristic mass fragments of *m/z* of 119.05 [C_8_H_6_O] + H^+^, 153.02 [C_7_H_4_O_4_] + H^+^, 253.14 [C_15_H_9_O_4_]^+^ followed by 3 3′ 4′ 5-tetrahydroxy-7-methoxy flavone (64) at *m/z* of 317.11 for [M + H]^+^. Myricetin (25) is the only aglycone identified in negative mode at *m/z* of 317.06 [M − H]^−^.

The flavonoids glycosides were detected in both negative and positive ion modes, these metabolites represented by 20 phytochemicals (12, 16, 20, 21, 29, 39, 45) in the negative ion mode at *m/z* of 461.16, 623.22, 507.17, 435.22, 463.11, 577.26, 609.15, respectively. While the remaining identified glycosides were (11, 18, 19, 20, 24, 27, 28, 32, 33, 37, 40, 44, 58, 60) in the positive ion mode at *m/z* of 593.28, 447.16, 449.14, 507.17, 465.10, 611.15, 417.13, 433.11, 593.17, 609.17, 595.37, 449.14, 433.12, 435.14, respectively. The major recognized glycosides were apigenin-7-*O*-glucoside (32) at (*m/z* 433.11) followed by luteolin-3′, 7-di-*O*-glucoside (27) at *m/z* 611.15 in the positive ion mode.

A total of 3 biflavonoids detected in ELME were procyanidin B1 (13) and B2 (23) at *m/z* 579.14 and 579.18 in positive mode, while procyanidin C1 (31) at *m/z* 865.21 in negative mode. Only two catechines were detected in ELME, 3 3′ 4′ 5 7-pentahydroxyflavan (17) and epicatechin (62) in the positive ion mode with [M + H]^+^ at *m/z* of 291.08 and 291.07. 

#### 3.1.2. Identification of Anthocyanidin-*O*-Glycosides

A total of 5 anthocyanidin glycoside derivatives were identified in ELME (26, 30, 35, 43, 61) in positive ion mode [M]^+^ at *m/z* 449.10, 479.11, 463.12, 611.22, 581.13, respectively. According to spectrum peak area, Cyanidin-3-*O*-2″-*O*-*β*-xylopyranosyl-*β*-glucopyranoside (61) was the major anthocyanidin glycoside detected in the ELME ([M]^+^ at *m/z* 581.13), followed by cyanidin-3-*O*-glucoside (26) [M]^+^ at *m/z* of 449.10.

#### 3.1.3. Identification of Carboxylic or Fatty, or Phenolic Acids Derivatives

Rosmarinic acid (2) and malic acid (3) were detected in ELME in the negative ion mode [M − H] at *m/z* 359.11 and 133.103. At the same time, 4-hydroxyphenyl-prop-2-enoic acid (5), chlorogenic acid (14), and methyl dihydrojasmonate (56) were identified in the positive ion mode with [M + H]^+^ at *m/z* 165.07, 355.07, and 227.16. The detected fatty acids were citraconic acid (1) in the negative ion mode [M − H] at *m/z* 129.02 and linoleic acid (10) in the positive ion mode with [M + H]^+^ at *m/z* 281.10.

#### 3.1.4. Identification of Alkaloids Derivatives

A total of 6 alkaloids’ derivatives were detected in the positive ion mode (7, 8, 9, 22, 49, 59) with [M + H]^+^ at *m/z* 215.05, 124.03, 192.10, 308.18, 195.13, 306.20, respectively. The highest one detected was harmaline (7) with [M + H]^+^ at *m/z* of 215.05, followed by dihydrocapsaicin (22) at *m/z* 308.18.

#### 3.1.5. Identification of Coumarins Derivatives

A total of 3 coumarins were detected in the positive ion mode, and they were 6,7-dihydroxy coumarin (15), daphnetin (36), and esculin (63). The highest one detected was daphnetin with [M + H]^+^ at m/z of 179.03 and characteristic mass fragments of *m/z* 77.03 [C_6_H_4_] + H^+^, 104.99 [C_6_H_3_O_2_-2H]^+^, 123.00 [C_6_H_4_O_3_-H]^+^, 133.02 [C_8_H_6_O_2_-H]^+^, 135.04 [C_8_H_6_O_2_] + H^+^, 151.03 [C_8_H_6_O_3_] + H^+^.

#### 3.1.6. Identification of Other Derivatives

Other detected compounds in the positive ion mode were 3-amino-1,2,4-triazole (*m/z* 85.027), resveratrol (6) at *m/z* 229.15, and monoterpenoids sabinene (34) *m/z* 137.05. Maritimetin-6-*O*-glucoside (38) from aurone derivatives was also detected with [M + H]^+^ at *m/z* 449.16. 

### 3.2. In Vitro Antifungal Activity of ELME

Using a disc agar diffusion method, we performed preliminary screening of the antifungal activity of ELME against *C. albicans* clinical isolates. We found that ELME exhibited antifungal activity as it resulted in the formation of inhibition zones around the disks. Thus, in the following step, we determined its MIC values using broth microdilution method. ELME had MIC values that ranged from 32–256 µg/mL, as shown in Table S2. 

### 3.3. In Vitro Antibiofilm Activity of ELME

Next, we assessed the antibiofilm activity of ELME (at 0.5 MIC values) as this virulence factor has a significant role in the development and spread of the infections caused by *C. albicans*. As shown in Table 2, ELME decreased the percentages of *C. albicans* fungal isolates that form biofilms moderately and strongly from 62.5% to 25%. 

#### 3.3.1. Biofilm Viability

**Figure 1:**
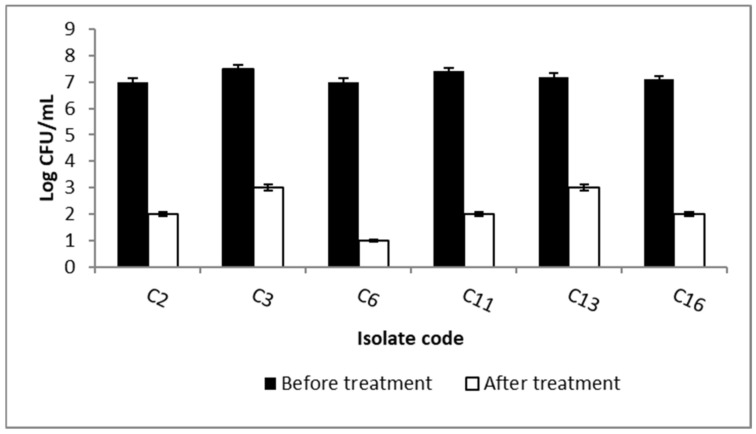
Bar chart revealing a significant reduction in the count of CFU/mL of the biofilm-forming *C. albicans* isolates after treatment with ELME.

To further study the antibiofilm activity of ELME, we assessed the viability of biofilms by counting the number of CFU/mL. Interestingly, we observed a significant reduction (*p* < 0.05) in the viability of the formed biofilms in 37.5% of the isolates by ELME, as shown in Figure 1. 

#### 3.3.2. SEM Examination

**Figure 2:**
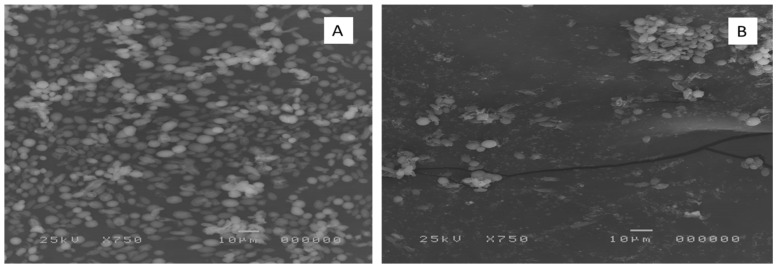
Scanning electron micrograph of the biofilm formed by a representative *C. albicans* isolate (C6) (**A**) before and (**B**) after treatment with ELME.

The impact of ELME on the biofilm formed by the tested *C. albicans* isolates was visually confirmed using SEM. As shown in the representative example in Figure 2, SEM was used to investigate the antibiofilm activity of ELME on the isolates, which showed a decrease in their biofilm-forming ability from moderately or strongly biofilm-forming to weakly or non-biofilm forming. ELME resulted in a significant reduction in biofilm formation. This finding is consistent with the detected inhibitory effect of ELME on biofilm by crystal violet assay and the biofilm viable cells count assay. 

#### 3.3.3. Fluorescent Microscope Examination

**Figure 3:**
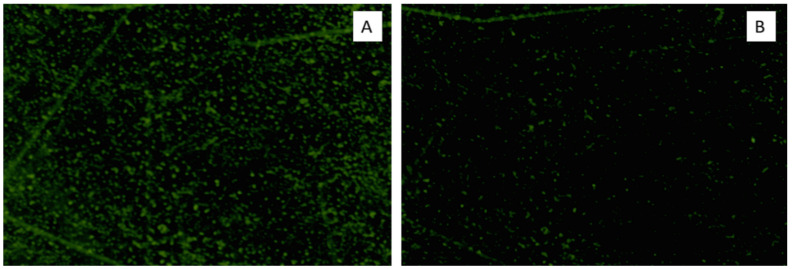
Fluorescent microscope micrograph of the biofilm formed by a representative *C. albicans* isolate (C6) stained with Calcofluor White stain (**A**) before and (**B**) after treatment with ELME.

A fluorescent microscope was used to investigate the antibiofilm activity of ELME on the isolates, which showed a decrease in their biofilm-forming ability from moderately or strongly biofilm-forming to weakly or non-biofilm forming. We found that ELME exhibited a significant reduction in biofilm formation, as revealed in Figure 3. A finding that could be correlated to the results of the examination of the effect of ELME on the formed biofilms by the tested *C. albicans* isolates using SEM. 

#### 3.3.4. qRT-PCR

**Figure 4:**
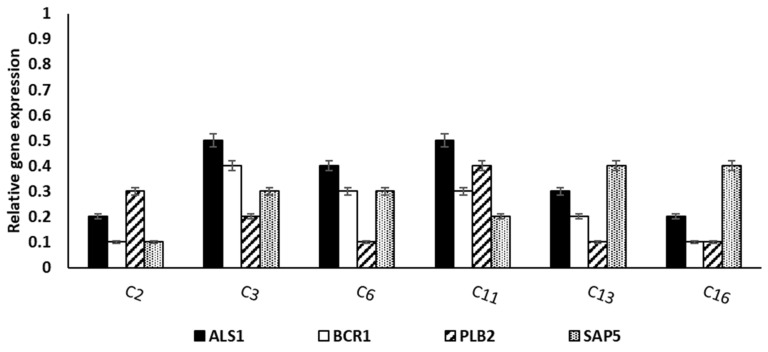
Bar chart showing the significant reduction in the relative expression of the biofilm genes in 6 (37.5%) *C. albicans* isolates after treatment with ELME.

It was carried out to elucidate the impact of ELME on the gene expression of the biofilm genes (*ALS1*, *BCR1*, *PLB2*, and *SAP5*). Interestingly, we observed a significant reduction in the expression of the biofilm genes in 37.5% of the tested *C. albicans* isolates, as shown in Figure 4. 

### 3.4. In Vivo Antibiofilm Activity of ELME

#### 3.4.1. Histopathological Examination Using H&E Stain

**Figure 5:**
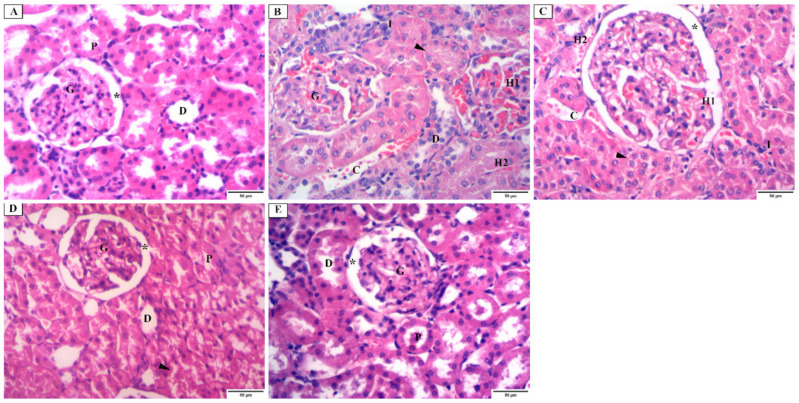
Photomicrograph of H&E stain of the renal cortex showing: (**A**) the control group (group I) having a renal corpuscle with a glomerulus (G), surrounded with parietal and visceral layers of bowman’s capsule, they are separated by bowman’s space (*, proximal convoluted tubules (P) are lined with pyramidal cells which have brush border, deeply acidophilic cytoplasm, and vesicular nuclei. Distal convoluted tubules (D) have a wide lumen, and their lining cells have apical nuclei and less acidophilic cytoplasm. (**B**) C. albicans group (group II) having renal glomeruli (G) with narrow bowman’s space and intraglomerular hemorrhage (H1). Interstitial hemorrhage (H2), cellular infiltration (I), and congested blood vessels (C) are seen. Proximal convoluted tubules with an obliterated lumen (arrowhead) and disrupted distal convoluted tubules (D) are noticed. (**C**) fluconazole group (group III) having renal glomeruli with apparent dilated bowman’s space (*) and apparent partial improvement from the previous group. Intraglomerular hemorrhage (H1), interstitial hemorrhage (H2), cellular infiltration (I), and congested blood vessels (C) are still seen. Proximal convoluted tubules with the obliterated lumen (arrowhead) are still noticed. (**D**) ELME treated group, 50 mg/kg (group IV), having segmented renal glomeruli with apparently normal bowman’s space (*). Proximal (P) and distal (D) convoluted tubules are nearly similar to the control group, but some tubules are disrupted with vacuolated cytoplasm in the tubular cells (arrowhead). (**E**) ELME treated group, 100 mg/kg (group V) have renal glomerulus (G), bowman’s space (*), proximal convoluted tubules (P), distal convoluted tubules (D), more or less similar to the control group (×200).

H&E-stained sections of the renal cortex of group I (control group) revealed the normal renal corpuscle, which contained glomerular capillaries and was surrounded by parietal and visceral layers of bowman’s capsule. Where bowman’s space separated the two layers, the parietal layer formed a simple squamous epithelium. Moreover, the proximal convoluted tubules (PCT) lined with pyramidal cells with deeply acidophilic cytoplasm and vesicular nuclei were observed. Distal convoluted tubules (DCT) showed a wide lumen, and its lining cells had apical nuclei and less acidophilic cytoplasm (Figure 5A). Sections of the renal cortex of group II (*C. albicans* group) exhibited glomerular and tubular changes. Renal glomeruli with narrow bowman’s space, intraglomerular hemorrhage, interstitial hemorrhage, cellular infiltration, and congested blood vessels were observed. The obliterated lumen and disrupted DCT were noticed (Figure 5B). Sections of the renal cortex of group III (fluconazole group) showed renal glomeruli with dilated bowman’s space and apparent partial improvement relative to the previous group. Intraglomerular hemorrhage, interstitial hemorrhage, cellular infiltration, and congested blood vessels were still seen (Figure 5C). Sections of the renal cortex of group IV (ELME treated group, 50 mg/kg) showed segmented renal glomeruli with apparently normal bowman’s space. PCT and DCT were nearly similar to the control group, but some tubules are disrupted with vacuolated cytoplasm in the tubular cells (Figure 5D). Sections of the renal cortex of group V (ELME treated group, 100 mg/kg) showed renal glomeruli, bowman’s space, PCT, and DCT are more or less similar to the control group (Figure 5E).

#### 3.4.2. Masson Trichrome Stain

**Figure 6:**
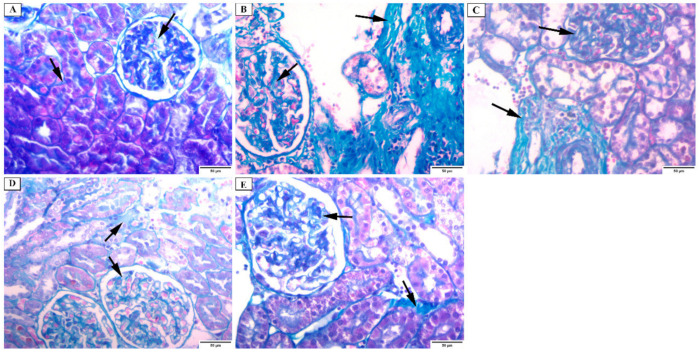
Photomicrograph of the renal cortex of Masson Trichrome stain showing: (**A**) group I have a minimal amount of collagen fibers in the renal interstitium and in between the glomerular capillaries (arrows). The basal lamina is positively stained. (**B**) group II has a massive increase in the collagen fibers in the interstitium and in between the glomerular capillaries (arrows). (**C**) group III has an intense increase in collagen fibers in the interstitium and between the glomerular capillaries (arrows). (**D**) group IV has a moderate increase in the collagen fibers in the interstitium and in between the glomerular capillaries (arrows). (**E**) group V has a mild increase in the collagen fibers in the interstitium and in between the glomerular capillaries (arrows) (×200).

Masson Trichrome stained kidney sections of group I (control) showed the presence of a minimal amount of collagen fibers in the interstitium and in between the glomerular capillaries. The basal lamina was positively stained (Figure 6A). In *C. albicans* group (group II), collagen fibers were massively increased in the interstitium and in between the glomerular capillaries (Figure 6B). However, collagen fibers intensely increased in the fluconazole group (group III) (Figure 6C). The renal cortex of the ELME treated group, 50 mg/kg (group IV), showed a moderate increase in the collagen fibers in the interstitium and between the glomerular capillaries (Figure 6D). While renal cortex of ELME treated group, 100 mg/kg (group V) showed a mild increase in the collagen fibers in the interstitium and between the glomerular capillaries (Figure 6E).

#### 3.4.3. Immunohistochemical Investigations

a. Desmin immunostaining

**Figure 7:**
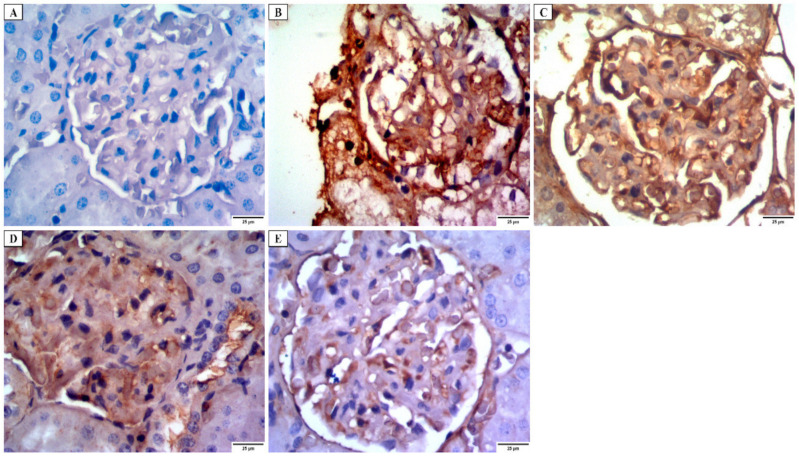
Photomicrograph of desmin immunostaining of the glomerular epithelial cells in the renal cortex showing: (**A**) group I have a faint positive cytoplasmic desmin immunostaining. (**B**) group II has a strong positive cytoplasmic desmin immunostaining. (**C**) group III has a moderately positive cytoplasmic desmin immunostaining. (**D**) group IV has a mild positive cytoplasmic desmin immunostaining. (**E**) group V has a weakly positive cytoplasmic desmin immunostaining (×400).

Kidney tissues obtained from group I showed a faint positive desmin expression in the glomerular epithelial cells (Figure 7A). Group II revealed strong positive cytoplasmic desmin immunostaining in the glomerular epithelial cells (Figure 7B). While group III revealed a moderate positive cytoplasmic desmin expression (Figure 7C). Group IV revealed a mild positive cytoplasmic desmin expression (Figure 7D). Group V showed a weak positive cytoplasmic desmin expression (Figure 7E).

b. Alkaline phosphatase enzyme immunostaining

**Figure 8:**
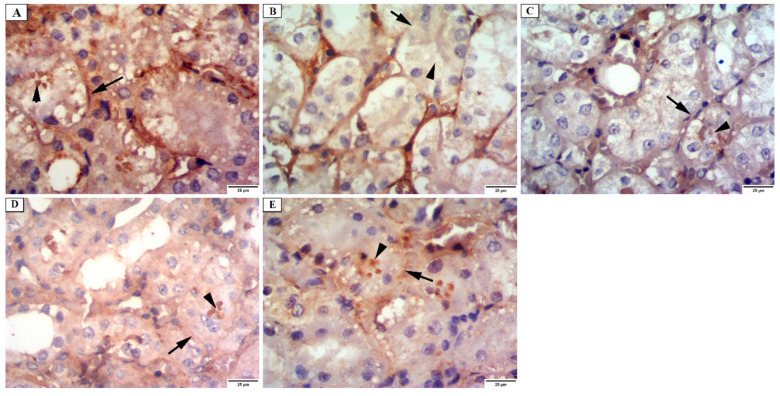
Photomicrograph of the renal cortex of alkaline phosphatase immunostaining showing: (**A**) group I have a strong positive reaction at the apical surfaces (arrowhead) and basal parts of the proximal convoluted tubular cells (arrow). (**B**) group II has a weak reaction at the apical surfaces (arrowhead) and basal parts of the proximal convoluted tubular cells (arrow). (**C**) group III has a mild positive reaction at the apical surfaces (arrowhead) and basal parts of the proximal convoluted tubular cells (arrow). (**D**) group IV has a moderate positive reaction at the apical surfaces (arrowhead) and a mild reaction in the basal parts of the proximal convoluted tubular cells (arrow). (**E**) group V has a strong positive reaction at the apical surfaces (arrowhead) and basal parts of the proximal convoluted tubular cells (arrow) (×400).

Regarding the alkaline phosphatase enzyme reaction in the examined groups, group I displayed a strong positive response at the apical surfaces and the basal parts of PCT (Figure 8A). A weak reaction was observed on the examination of group II (Figure 8B). Sections of the renal cortex of group III displayed a mild positive reaction (Figure 8C). Sections of group IV showed a moderately positive reaction (Figure 8D). While sections of group V showed a strong positive reaction for alkaline phosphatase (Figure 8E).

c. iNOs immunostaining

**Figure 9:**
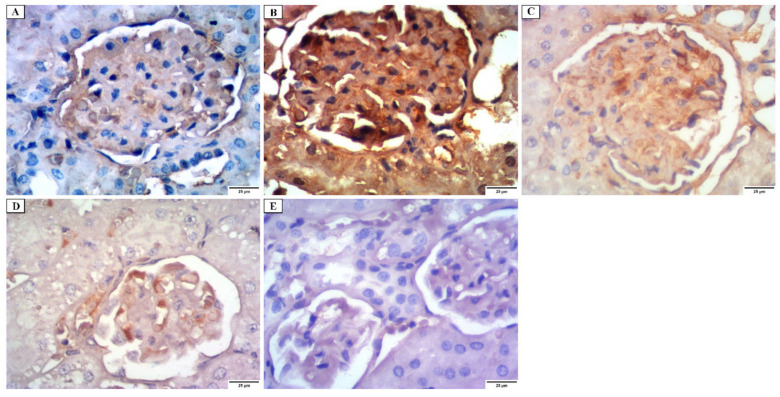
Photomicrograph of the renal cortex of iNOs immunostaining showing: (**A**) group I have a weakly positive reaction at the cytoplasm of glomeruli capillary endothelium cells and a faint positive reaction in the cytoplasm of the tubular cells. (**B**) group II has a strong positive reaction at the cytoplasm of glomeruli capillary cells and tubular cells. (**C**) group III has a moderately positive reaction at the cytoplasm of glomeruli capillary cells and tubular cells. (**D**) group IV has a mild positive reaction at the cytoplasm of glomeruli capillary cells and tubular cells. (**E**) group V has a faint positive reaction at the cytoplasm of glomeruli capillary cells and tubular cells (×400).

Immunohistochemical staining for iNOs immunoreaction of the control group revealed faint positive immunoreaction in the cytoplasm of the renal tubular cells. A weak positive immunoreaction in the cytoplasm of the glomerular capillary endothelium was also observed (Figure 9A). Group II showed a strong positive reaction in the cytoplasm of the glomeruli capillary cells and the tubular cells (Figure 9B). While group III showed a moderately positive reaction in the cytoplasm of the glomeruli capillary cells and the tubular cells (Figure 9C), group IV showed a mild positive reaction in the cytoplasm of the glomeruli capillary cells and the tubular cells (Figure 9D). Group V showed a faint positive reaction in the cytoplasm of glomeruli capillary cells and the tubular cells (Figure 9E).

#### 3.4.4. Morphometric Analysis

**Figure 10:**
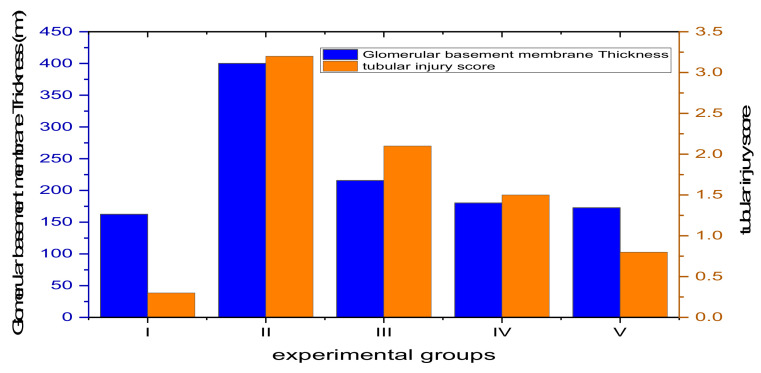
Bar chart showing the thickness of the glomerular basement membrane ((**left**) axis) and the tubular injury score ((**right**) axis) among the experimental groups.

As shown in Figure 10 and Table S3, group II exhibited a highly significant increase in the thickness of the glomerular basement membrane in comparison with the control group (*p* ˂ 0.001). Group III displayed a substantial increase in thickness compared to the control group (*p* ˂ 0.05). On the other hand, groups IV and V showed a non-significant change (*p* > 0.05) compared to the control group. In addition, group II exhibited a significant increase (*p* ˂ 0.001) in the tubular injury score compared to the control group. Group III showed a substantial increase in the tubular injury score compared to the control group (*p* ˂ 0.05). Meanwhile, groups IV and V showed a non-significant change (*p* > 0.05) when compared to the control group (Figure 10). 

**Figure 11:**
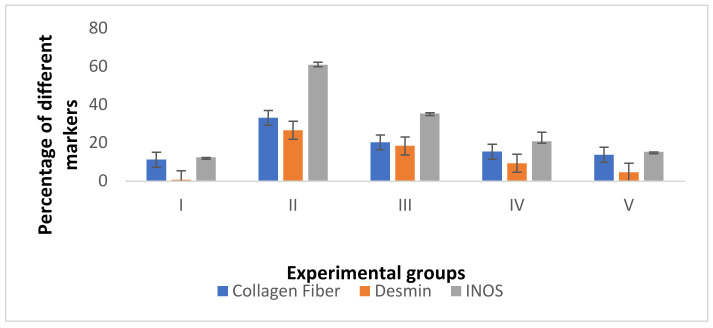
Bar chart showing the percentages of collagen fiber, desmin, and iNOs among the different groups.

As presented in Figure 11 and Table S4, group II exhibited a highly significant increase (*p* ˂ 0.001) in the percentages of collagen, desmin, and iNOs when compared to the control group. However, groups III and IV showed a significant increase (*p* ˂ 0.05) in the percentages of desmin and iNOs compared to control. On the other side, groups IV and V exhibited a non-significant collagen change (*p* > 0.05) in comparison with the control group. Moreover, group V showed a non-significant desmin and iNOs increase (*p* > 0.05) compared to the control group.

#### 3.4.5. Counting the CFU/g and Determination of the Survival Rates

**Figure 12:**
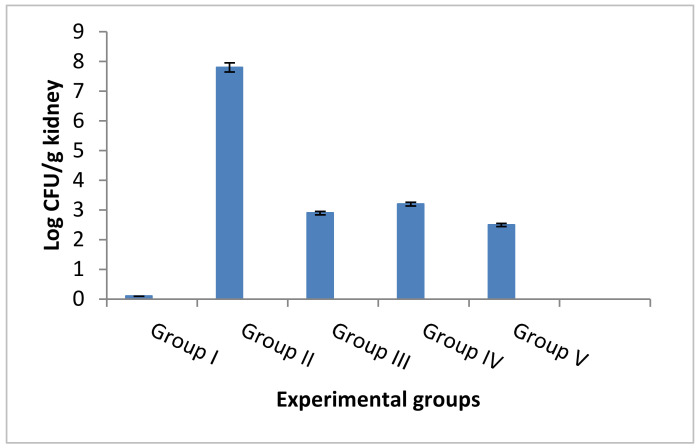
Bar chart showing the number of CFU/g kidney of the rats of different experimental groups.

The number of CFU/g kidney tissues was counted in the tested five experimental groups. ELME resulted in a significant reduction in the number of CFU/g kidneys in the infected rats, as shown in Figure 12.

**Figure 13:**
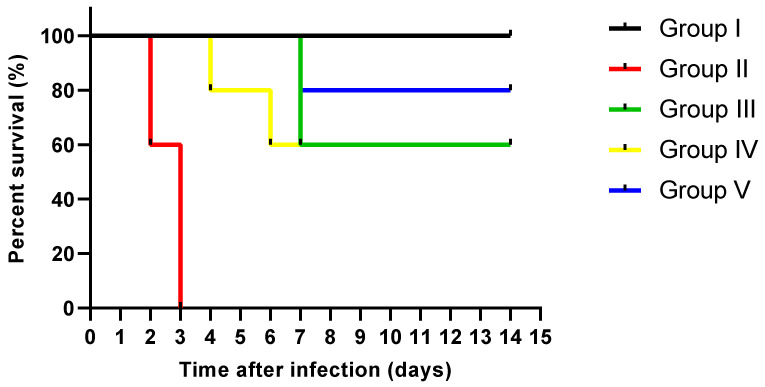
Survival of rats with systemic candidiasis using Kaplan-Meier survival curve.

A total of 5 rats were left in each group for observing their survival rate for 14 days. As shown in Figure 13, all rats in the control group (group I) were alive till the last day of the experiment. On the other hand, two rats in *C. albicans* group (group II) died after two days, and three rats died after three days. In the fluconazole treated group (group III), two rats died after one week, and three remained alive until the end of the experiment. In the ELME treated group, 50 mg/kg (group IV), only 1 rat died after 4 days, and another one died after 6 days while the other rats remained alive to the end of the experiment. In ELME treated group, 100 mg/kg (group V), only 1 rat died after 7 days, and the rest were alive until the end of the experiment.

### 3.5. Cytotoxicity MTT Assay

The cytotoxicity of ELME was investigated on HSF normal cells using MTT assay. In comparison with doxorubicin as a positive control, which had an IC_50_ = 4.42 µg/mL, ELME had an IC_50_ of 31.26 µg/mL, as shown in Figure S3.

## 4. Discussion

Fungal infections are spreading widely on a global scale, affecting more than 1 billion people worldwide, resulting in high morbidity and mortality rates. This is in addition to high medical costs. Despite the relative availability of antifungal drugs, the fatality rates among patients infected with candidiasis are very high [32]. Thus, there is a great need for new antifungals with high activity against pathogenic fungi, particularly *C. albicans.* Plants are a valuable source for antimicrobials; therefore, we investigated the antifungal activity of ELME against *C. albicans* clinical isolates using adisc agar diffusion method as a preliminary test for screening the antimicrobial activity. Then, using broth microdilution method, ELME exhibited MIC values that ranged from 32–256 µg/mL. Several researchers worldwide have examined the antifungal activity of many plant extracts to solve the problem of the dissemination of resistance among fungal isolates [33,34,35]. Some of them have used the total plant extracts, whilst others have investigated the activity of one bioactive compound from the plant. Diba and Alizadeh [36], for example, examined the antifungal activity of *Allium hirtifolium* and *Allium sativum* plant extracts. On the other hand, Alalwan et al. [37] and Zarrinfar et al. [9] investigated the antifungal activity of curcumin against different fungi. 

*Candida* biofilms are usually formed in the human body in mucosa or endothelium, leading to the development of candidiasis. In addition, they are associated with medical devices such as catheters. Biofilm formation by *C. albicans* is highly linked to resistance development to the commonly used antifungal drugs [38]. Therefore, we investigated the antibiofilm activity of ELME against the biofilms formed by *C. albicans* isolates. Interestingly, ELME displayed antibiofilm activity as it decreased the percentages of *C. albicans* isolates moderately and strongly biofilm forming from 62.5% to 25%. Herein, we chose the CFU method to assess the viability of the biofilm-forming isolates before and after treatment with ELME. It is noteworthy that the CFU method could differentiate between the living and dead cells as the viable cells only are counted with the exclusion of the dead ones and other debris [39]. The current investigation revealed that ELME resulted in a significant reduction in the viability of the formed biofilms in 37.5% of *C. albicans* isolates. 

Unfortunately, the research scope in antifungals is not that of the antibacterial owing to the lower number of infections caused by fungi when compared to bacteria. Moreover, fungi are eukaryotes, similarly to humans, so it is important to the antifungal compound to be non-toxic to human cells. However, some studies have investigated the antifungal and antibiofilm activities of plant extracts against *C. albicans*, especially in oral diseases [40,41]. 

The impact of ELME on the biofilm morphology was elucidated using SEM and a fluorescent microscope. A significant reduction in the biofilm formation by *C. albicans* isolates was observed, confirming the results of crystal violet and CFU assays. Moreover, the gene expression of the genes related to biofilm formation was examined in the treated isolates. We observed that ELME resulted in the downregulation of the tested genes in 37.5% of the isolates. Biofilm formation by *C. albicans* is determined by many transcription factors such as *ALS1*, *BCR1*, *PLB2*, and *SAP5* [42]. Such transcription factors have an essential function in several pathways in the fungal cells, and they could influence the adherence of *C. albicans* isolates [42]. 

Few studies investigated the in vivo antifungal activity of plant extract especially on kidney. Thus, we tried in this study to elucidate the potential protective effect of ELME against *C. albicans* on kidney tissues. In our in vivo experiment, *C. albicans* group showed damaged glomeruli and tubules accompanied by hemorrhage, cellular infiltration, and congested blood vessels. This could be explained by the finding reported by Zhao et al. [43]. They stated that yeasts pass through the vascular walls attracting neutrophilic infiltration and causing an inflammatory reaction confirmed by a highly significant response with iNOs. 

Inflammation interferes with the antioxidant defense mechanism, leading to reactive oxygen species generation and renal glomeruli and tubules damage [44]. This was confirmed by morphometric and immunohistochemical results of *C. albicans* group, while the fluconazole treated group showed partial improvement.

The group treated with 50 mg/kg ELME showed glomeruli and tubules nearly similar to the normal control, but some tubules were disrupted. While the group treated with 100 mg/kg ELME showed glomeruli and tubules more or less similar to the control group. These findings are in harmony with morphometric and immunohistochemical results. ELME was found to have protective effects on the kidney tissues as the amount of collagen fibers in the renal interstitium and between the glomerular capillaries decreased. In addition, it increased the level of alkaline phosphatase, an enzyme having anti-inflammatory effects, and it reduced the levels of iNOs and desmin, which are highly associated with inflammation and oxidative stress [13]. 

LC-MS/MS of ELME identified 64 molecules belonging to several phytochemical subclasses. This investigation revealed that ELME possesses anti-inflammatory effects which may be ascribed to several active constituents present in, such as flavonoids, biflavonoids and their glycosides derivatives [45,46,47], alkaloids [48], aurone [49], coumarins [50], and phenolic acid [51,52]. ELME included various alkaloids, flavonoids, and phenolic compounds, such as harmaline, dihydrocapsaicin, luteolin, kaempferol, and isorhamnetin derivatives, which were believed to block NO generation and explain ELME’s anti-inflammatory, antioxidant, and antifungal properties [53,54,55,56].

The nature and quantity of active constituents might vary during different stages of leaves development so this could affect the activity of the plant. Moreover, it would also be interesting to evaluate the content and activity of the leaves collected in different stages of development in further studies.

Our findings align with prior research on the antimicrobial properties of certain flavonoids or flavonoid glycosides, such as myricetin [57,58], apigenin derivatives [59], luteolin derivatives [60], epicatechin [57], anthocyanidin derivatives [61,62], biflavonoids derivatives [13,14,63,64], coumarines derivatives [65,66], harmaline alkaloid [67].

## 5. Conclusions

In the present work, the phytoconstituents of the *E. laurentianus* De Wild. leaves methanol extract were determined using LC-MS/MS for the first time. Altogether, 64 compounds were identified, including anthocyanidin-3-*O*-glycosides, alkaloids, aurone derivative, coumarins, flavonoid aglycone, flavonoid glycoside, phenolic or organic acids.

Our results demonstrate the antifungal and antibiofilm activities of ELME against *C. albicans* clinical isolates in vitro using different techniques such as examination with SEM and fluorescent microscope as well as qRT-PCR In addition, the in vivo study revealed that ELEM could protect against renal cortical damage induced by *C. albicans* in an albino rat model using histological and immunohistochemical investigations. This could have occurred via the inhibition of oxidative stress and inflammation. Still, further studies are required to explore the exact mechanisms underlying the effects of ELEMI as an antifungal drug.

## Figures and Tables

**Table 1:** Phytochemical profiling of ELME by LC-MS/MS analysis (negative and positive mode E.S.I.).

Peak NO	Identification	Error	R.T. (min.)	*m*/*z*	Adduct Ion	Formula	MS/MS
1	^f.a^ Citraconic acid	−2.8	1.006	129.02	[M − H]^−^	C_5_H_6_O_4_	84.99, 85.02, 129.01
2	^a^ Rosmarinic acid	6.0	1.009	359.11	[M − H]^−^	C_18_H_16_O_8_	181.04, 329.09, 359.07, 359.11
3	^a^ Malic acid	−4.7	1.331	133.10	[M − H]^−^	C_4_H_6_O_5_	133.01
4	^t^ 3-Amino-1,2,4-triazole	−0.6	1.392	85.027	[M + H]^+^	C_2_H_4_N_4_	71.95, 85.02
5	^a^ 3-(4-hydroxyphenyl)prop-2-enoic acid	−0.5	1. 393	165.07	[M + H]^+^	C_9_H_8_O_3_	61.02, 85.02, 166.08
6	^s^ Resveratrol	6.4	1.470	229.15	[M + H]^+^	C_14_H_12_O_3_	58.06, 60.08, 70.06, 229.15
7	^ak^ Harmaline	0.6	1.561	215.05	[M + H]^+^	C_13_H_14_N_2_O	72.08, 156.10, 169.13, 215.05
8	^ak^ Nicotinic acid	−0.9	1.693	124.03	[M + H]^+^	C_6_H_5_NO_2_	53.03, 78.03, 80.04, 124.03
9	^ak^ 5-Hydroxyindoleacetic acid	0.8	1.3058	192.10	[M + H]^+^	C_10_H_9_NO_3_	148.07, 177.07, 192.10
10	^f.a^ Linoleic acid	0.6	2.290	281.10	[M + H]^+^	C_18_H_32_O_2_	123.07, 151.04, 165.09, 281.05
11	^f.g^ Acacetin-7-*O*-neohesperidoside	−8.1	4.305	593.28	[M + H]^+^	C_28_H_32_O_14_	593.25
12	^f.g^ Kaempferol-3-Glucuronide	10.1	5.108	461.16	[M − H]^−^	C_21_H_18_O_12_	188.93, 239.09, 256.92, 324.90, 392.89, 461.15
13	^b^ Procyanidin B1	1.5	5.115	579.14	[M + H]^+^	C_30_H_26_O_12_	123.04, 127.03, 135.04, 579.14
14	^a^ Chlorogenic acid	1.3	5.534	355.07	[M + H]^+^	C_16_H_18_O_9_	135.04, 163.04, 355.07
15	^co^ 6,7-dihydroxycoumarin	−0.4	5.439	179.03	[M + H]^+^	C_9_H_6_O_4_	51.02, 77.03 179.03
16	^f.g^ Isorhamnetin-3-*O*-rutinoside	−2.1	5.465	623.22	[M − H]^−^	C_28_H_32_O_16_	315.03, 532.91, 579.15, 623.17
17	^c^ (+)-3 3′ 4′ 5 7-Pentahydroxyflavan	3.6	5.574	291.08	[M + H]^+^	C_15_H_14_O_6_	111.04, 119.04, 123.04, 291.08
18	^f.g^ Baicalein-7-*O*-glucuronide	4.2	5.575	447.16	[M + H]^+^	C_21_H_18_O_11_	125.13, 129.07, 135.04, 269.06, 273.08, 447.13
19	^f.g^ Luteolin-8-C-glucoside	−12.6	5.656	449.14	[M + H]^+^	C_21_H_20_O_11_	139.07, 449.12
20	^f.g^ Syringetin-3-*O*-galactoside	−5.2	5.693	507.17	[M − H_2_O − H]^−^	C_23_H_24_O_13_	112.98, 138.02, 163.06, 218.95, 286.93, 307.10, 354.92, 507.16
21	^f.g^ Phlorizin	2.7	5.707	435.22	[M − H]^−^	C_21_H_24_O_10_	389.21, 433.83, 435.20, 435.23
22	^ak^ Dihydrocapsaicin	3.3	5.974	308.18	[M + H]^+^	C_18_H_29_NO_3_	123.04, 131.04, 137.05, 149.05
23	^b^ Procyanidin B2	0.6	6.161	579.18	[M + H]^+^	C_30_H_26_O_12_	112.02, 579.18
24	^f.g^ Quercetin-4′-glucoside	−1.3	7.061	465.10	[M + H]^+^	C_21_H_20_O_12_	69.03, 115.04, 289.04, 303.04, 465.09
25	^f^ Myricetin	−1.5	7.063	317.06	[M − H]^−^	C_21_H_24_O_10_	112.98, 155.03, 165.98, 180.96, 194.99, 274.04, 287.08, 302.07
26	^an^ Cyanidin-3-glucoside	1.0	7.109	449.10	[M]^+^	C_21_H_21_O_11_	85.01, 117.02, 147.02, 201.02, 287.05, 449.10
27	^f.g^ Luteolin-3′,7-di-*O*-glucoside	−0.4	7.227	611.15	[M + H]^+^	C_27_H_30_O_16_	135.04, 148.10, 271.06, 273.06, 611.15
28	^f.g^ Daidzein-8-C-glucoside	−0.5	7.279	417.13	[M + H]+	C_21_H_20_O_9_	180.06, 399.21, 417.13
29	^f.g^ Isoquercitrin	−2.1	7.283	463.11	[M − H]^−^	C_21_H_20_O_12_	48.17, 462.90
30	^an^ Petunidin-3-*O*-beta-glucopyranoside	0.9	7.567	479.11	[M]^+^	C_22_H_23_O_12_	165.09, 211.12, 285.08, 299.10, 302.04, 479.11
31	^b^ Procyanidin C1	0.9	7.772	865.21	[M-H]-	C_45_H_38_O_18_	11.46, 865.20
32	^f.g^ apigenin-7-*O*-glucoside	3.5	7.891	433.11	[M + H]^+^	C_21_H_20_O_10_	119.04, 141.07, 148.11, 135.02, 229.04, 270.22, 433.11
33	^f.g^ Acacetin-7-*O*-rutinoside	8.0	7.870	593.17	[M + H]^+^	C_28_H_32_O_14_	520.20, 575.32, 593.17
34	^tp^ Sabinene	0.4	8.068	137.05	[M + H]^+^	C_10_H_16_	55.05, 66.04, 68.74, 79.05, 94.04, 137.05
35	^an^ Peonidine-3-*O*-glucoside chloride	−6.1	8.101	463.12	[M]^+^	C_22_H_23_O_11_	73.05,167.99, 197.47, 234.10, 258.04, 281.08, 286.04, 301.06, 309.47, 342.01, 399.15, 463.12
36	^co^ Daphnetin	−0.8	8.202	179.03	[M + H]^+^	C_9_H_6_O_4_	77.03, 104.99, 123.00, 133.02, 135.04, 151.038
37	^f.g^ Diosmin	−0.8	8.762	609.17	[M + H]^+^	C_28_H_32_O_15_	265.11, 303.11, 609.16
38	^au^ Maritimetin-6-*O*-glucoside	0.8	8.827	449.16	[M + H]^+^	C_21_H_20_O_11_	74.09, 133.02,135.04, 257.04, 285.03, 360.05, 375.07, 388.05, 403.07, 417.09, 434.09, 449.12
39	^f.g^ Rhoifolin	0.7	8.974	577.26	[M − H]^−^	C_27_H_30_O_14_	576.81, 532.90, 269.10
40	^f.g^ Apigenin-6-C-glucoside -7-*O*-glucoside	−2.0	9.027	595.37	[M + H]^+^	C_21_H_20_O_12_	165.02, 177.06, 285.09, 303.05
41	^f^ 3′ 4′ 5 7-tetrahydroxyflavanone	0.0	9.760	289.07	[M + H]^+^	C_15_H_12_O_6_	117.03, 121.06, 135.04, 139.03, 145.02, 153.01, 163.03, 179.03, 181.06, 289.07
42	^f^ Luteolin	8.1	10.002	287.04	[M + H]^+^	C_15_H_10_O_6_	67.01, 77.04, 287.04
43	^an^ Cyanidin-3, 5-di-*O*-glucoside	−1.2	10.357	611.22	[M]^+^	C_27_H_31_O_16_	215.06, 266.99, 309.06, 355.06, 449.13, 594.23, 611.22
44	^f.g^ Luteolin-4′-*O*-glucoside	−3.9	10.370	449.14	[M + H]^+^	C_21_H_20_O_11_	147.04, 153.06, 167.02, 287.05, 287.10, 449.14
45	^f.g^ Rutin	1.7	10.842	609.15	[M − H]^−^	C_27_H_30_O_16_	609.15
46	^f^ Naringenin	0.9	11.086	273.07	[M + H]^+^	C_15_H_12_O_5_	67.04, 111.08, 119.03, 125.10, 129.07, 135.01. 273.07
47	^f^ 3 5 7-trihydroxy-4′-methoxyflavone	0.7	11.278	301.10	[M + H]^+^	C_16_H_12_O_6_	181.06, 215.07, 223.07, 258.08, 273.11, 301.11
48	^f^ 3′-Methoxy-4′,5,7-trihydroxyflavonol	0.4	11.329	317.06	[M + H]^+^	C_16_H_12_O_7_	129.97, 137.02, 168.00, 245.04, 263.21, 274.04, 287.09, 302.04, 317.06
49	^ak^ Caffeine	−1.9	11.857	195.13	[M + H]^+^	C_8_H_10_N_4_O_2_	195.13
50	^f^ Formononetin	5.9	12.010	269.07	[M + H]^+^	C_16_H_12_O_4_	137.02, 225.06, 254.06, 269.07
51	^f^ 4′,5-dihydroxy-7-methoxyflavone	−2.6	12.613	287.09	[M + H]^+^	C_16_H_14_O_5_	137.02, 145.08, 167.03, 175.07, 287.09
52	^f^ Acacetin	1.0	12.776	285.08	[M + H]^+^	C_16_H_12_O_5_	128.06, 207.06, 241.04, 242.05, 270.05, 285.07
53	^f^ Apigenin	0.8	12.788	271.09	[M + H]^+^	C_15_H_10_O_5_	65.04, 67.01, 68.99, 89.03, 109.02, 115.05, 153.02, 163.04, 253.14, 271.09
54	^f^ 4′,5,7-Trihydroxyflavonol	0.9	14.059	287.09	[M + H]^+^	C_15_H_10_O_6_	67.02, 91.05, 111.04, 119.04, 124.01, 147.04, 167.03, 287.09
55	^f^ (+-)-Taxifolin	−0.1	14.290	305.13	[M + H]^+^	C_15_H_12_O_7_	305.13
56	^a^ Methyl dihydrojasmonate	−9.7	14.433	227.16	[M + H]^+^	C_13_H_22_O_3_	79.05, 95.08, 167.14, 195.14, 227.14
57	^f^ 3′ 4′ 5 7-tetrahydroxyflavanone	−1.4	14.553	289.18	[M + H]^+^	C_15_H_12_O_6_	271.17, 289.18
58	^f.g^ Apigenin 8-C-glucoside	−6.3	14.961	433.12	[M + H]^+^	C_21_H_20_O_10_	135.03, 391.09, 433.13
59	^ak^ Capsaicin	0	15.293	306.20	[M + H]^+^	C_18_H_27_NO_3_	108.04, 126.02, 137.06, 153.12, 306.20
60	^f.g^ Quercetin-3-Arabinoside	−6.8	15.368	435.14	[M + H]^+^	C_20_H_18_O_11_	135.04, 240.04, 271.06, 389.10, 435.14
61	^an^ Cyanidin-3-*O*-(2″-*O*-*β* -xylopyranosyl*-β*-glucooside)	8.6	17.917	581.13	[M]^+^	C_26_H_29_O_15_	107.04, 133.06, 135.04, 153.01, 297.07, 581.14
62	^c^ (-)-Epicatechin	2.5	18.280	291.07	[M + H]^+^	C_15_H_14_O_6_	81.07, 135.05, 275.05, 291.07
63	^co^ Esculin	−2.2	18.380	341.19	[M + H]^+^	C_15_H_16_O_9_	112.07, 121.14, 131.04, 139.08, 161.06, 165.08, 179.12, 180.13, 287.24, 341.19
64	^f^ 3 3′ 4′ 5-tetrahydroxy-7-methoxyflavone	−4.7	20.178	317.11	[M + H]^+^	C_16_H_12_O_7_	105.07, 129.07, 215.18, 267.20, 299.20, 317.11

^a^: carboxylic acid or phenolic acid derivative, ^f.a^: fatty acids, ^ak^: alkaloid and related metabolites, ^an^: anthocyanidin glycosides, ^au^: aurone derivative, ^c^: catechins ^co^: coumarins, ^f^: flavonoid aglycone, ^f.g^: flavonoid glycoside, ^b^: biflavonoids, ^s^: stilbenes, ^t^: triazoles ^tp^: terpenoids.

**Table 2:** Effect of ELME on the biofilm-forming ability of *C. albicans* isolates.

Biofilm Forming Ability	No. of Isolates before Treatment with ELME	No. of Isolates after Treatment with ELME
Non-biofilm forming	4	6
Weak biofilm-forming	2	6
Moderate biofilm-forming	6	3
Strong biofilm-forming	4	1

## Data Availability

Data is contained within the article and Supplementary Material.

## Supplementary Materials

The following are available online at https://www.mdpi.com/article/10.3390/jof8050426/s1, Figure S1: Total ion chromatogram by LC-MS/MS of ELME in positive ion mode, Figure S2: Total ion chromatogram by LC-MS/MS of ELME in negative ion mode, Figure S3: MTT cytotoxicity assay of ELME against HSF normal cell line. Table S1: Sequences of primer used in qRT-PCR, Table S2: Values of MICs of ELME against the tested *C. albicans* clinical isolates, Table S3: Means values of the glomerular basement membrane thickness, tubular injury score in different experimental groups, Table S4: Percentages of collagen, desmin and iNOs expression in different experimental groups.
