# Supplementary material for: Promising Antifungal Activity of Encephalartos laurentianus de Wild against Candida albicans Clinical Isolates: In Vitro and In Vivo Effects on Renal Cortex of Adult Albino Rats
Source: J Fungi (Basel). 2022 Apr 21;8(5):426. doi: 10.3390/jof8050426 (PMC9144060; doi:10.3390/jof8050426)
Supplement: Supplementary file 1 [file jof-08-00426-s001.zip › jof-1662771-supplementary.pdf]

Journal of Fungi

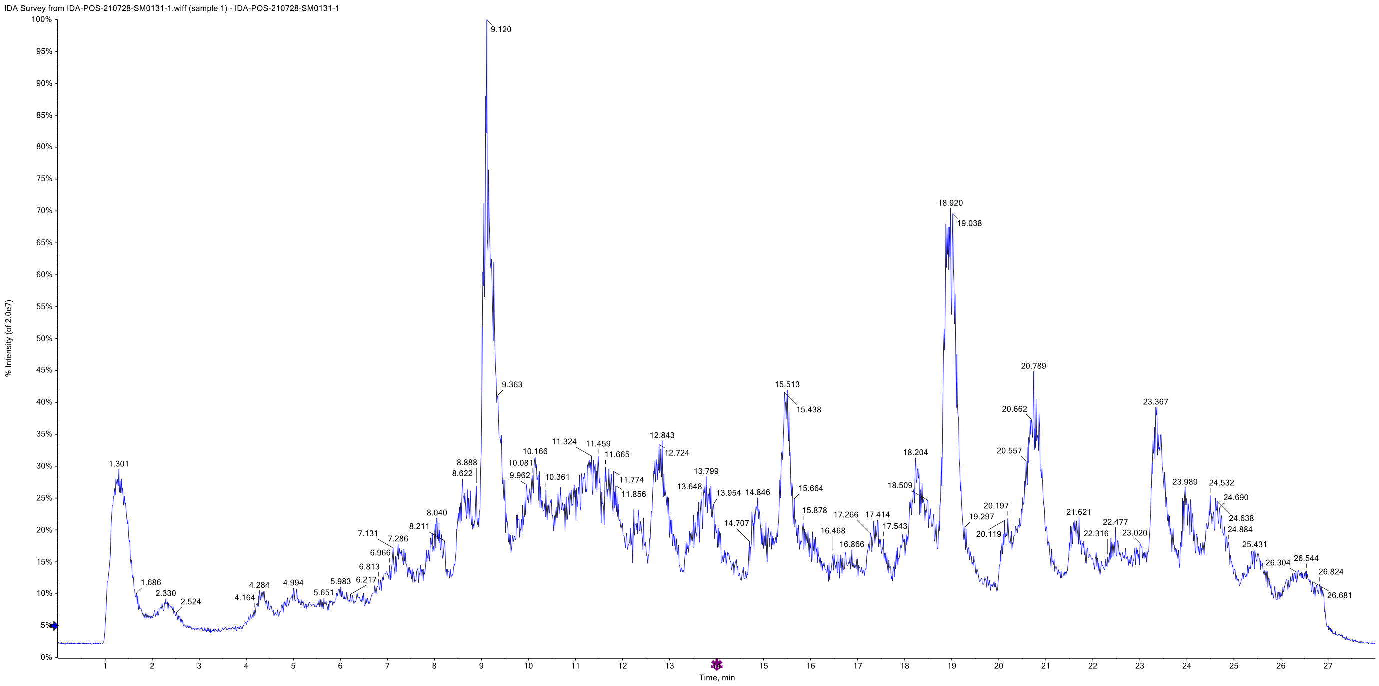

Figure S1. Total ion chromatogram by LC-MS/MS of ELME in positive ion mode.

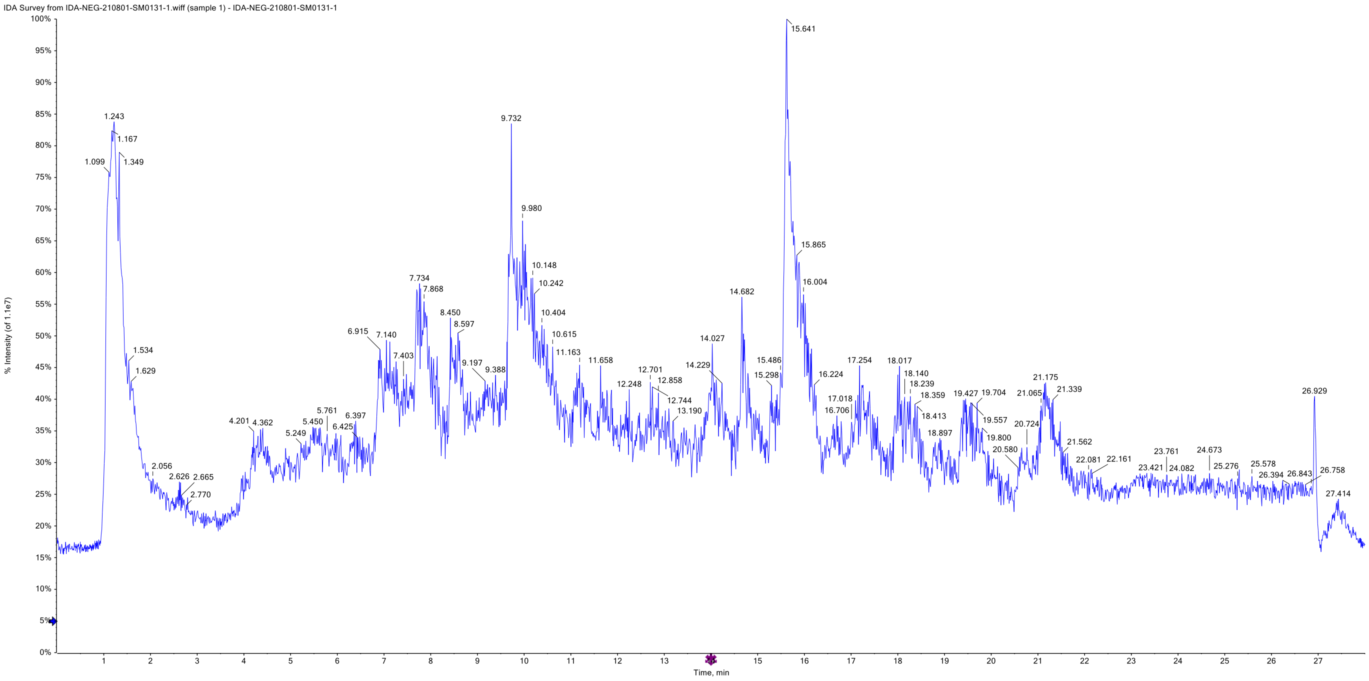

Figure S2. Total ion chromatogram by LC-MS/MS of ELME in negative ion mode.

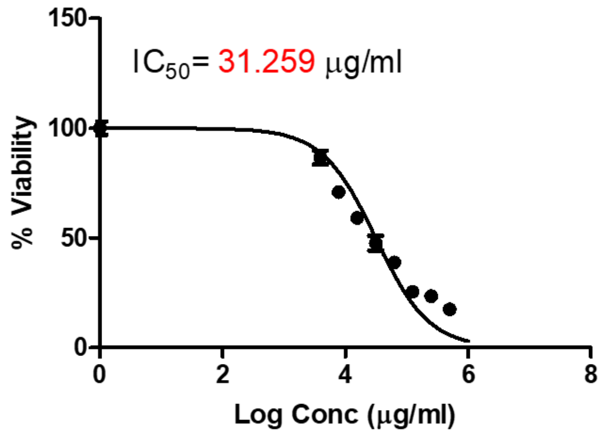

Figure S3. MTT cytotoxicity assay of ELME against HSF normal cell line.

Table S1. Sequences of primer used in qRT-PCR.

Gene name	Sequence 5′- 3′
ACT1 (reference gene)	F- TTTCATCTTCTGTATCAGAGGAACTTAT<br>R- ATGGGATGAATCATCAAACAAGAG
ALS1	F- CAACAGGCACCTCAGCATCTAC<br>R- CTCCACCAGTAACAGATCCACTAGTAA
BCR1	F- AATGCCTGCAGGTTATTTGG<br>R- TTTTAGGTGGTGGTGGCAAT
PLB2	F- TGAACCTTTGGGCGACAACT<br>R- GCCGCGCTCGTTGTTAA
SAP5	F- CCAGCATCTTCCCGCACTT<br>R- GCGTAAGAACCGTCACCATATTTAA

Table S2. Values of MICs of ELME against the tested *C. albicans* clinical isolates.

Isolate code	MIC (µg/mL)	Isolate code	MIC (µg/mL)
C1	32	C9	64
C2	32	C10	32
C3	64	C11	32
C4	32	C12	64
C5	64	C13	64
C6	32	C14	128
C7	128	C15	256
C8	128	C16	256

Table S3. Means values of the glomerular basement membrane thickness, tubular injury score in different experimental groups.

	I			II			III			IV			V		
**Glomerular basement membrane (nm)**	**162.5**	**±**	**7.2**	**400.1**	**±**	**11.9 ****	**215.6**	**±**	**5.7 ***	180.3	±	6.8	172.8	±	5.1
**Tubular injury score**	0.3		(0-2)	3.2		(1-5)**	2.1		(1-4)*	1.5		(0-3)	0.8		(0-2)

(**) Highly Significant increase compared to the control group (*p*<0.001). (*) Significant increase compared to control group (*p*<0.05).

Table S4. Percentages of collagen, desmin and iNOs expression in different experimental groups.

	I			II			III			IV			V		
**Collagen fiber %**	**11.21**	**±**	**1.36**	**33.12**	**±**	**1.56 ****	**20.3**	**±**	**1.96 ***	15.4	±	2.32	13.81	±	1.72
**Desmin %**	0.7	±	0.4	26.6	±	2.6**	18.4	±	1.7 *	9.3	±	1.8*	4.6	±	3.9
**iNOs %**	12.4	±	4.8	60.9	±	3.2 **	35.4	±	2.1 *	20.8	±	6.8*	15.3	±	2.9

(**) Highly Significant increase compared to the control group (*p*<0.001).(*) Significant increase compared to control group (*p*<0.05).
